# Single-CpG resolution mapping of 5-hydroxymethylcytosine by chemical labeling and exonuclease digestion identifies evolutionarily unconserved CpGs as TET targets

**DOI:** 10.1186/s13059-016-0919-y

**Published:** 2016-03-29

**Authors:** Aurélien A. Sérandour, Stéphane Avner, Elise A. Mahé, Thierry Madigou, Sylvain Guibert, Michaël Weber, Gilles Salbert

**Affiliations:** EMBL, Meyerhofstrasse 1, Heidelberg, 69117 Germany; CNRS UMR6290, Equipe SP@RTE, Institut de Génétique et Développement de Rennes, Campus de Beaulieu, Rennes cedex, 35042 France; Université de Rennes 1, Campus de Beaulieu, Rennes Cedex, 35042 France; CNRS, Université de Strasbourg, UMR7242, Biotechnologie et signalisation cellulaire, 300 bd Sébastien Brant, Illkirch cedex, 67412 France

**Keywords:** 5-hydroxymethylcytosine, 5hmC, Single-CpG resolution, Selective chemical labeling

## Abstract

Conventional techniques for single-base resolution mapping of epigenetic modifications of DNA such as 5-hydroxymethylcytosine (5hmC) rely on the sequencing of bisulfite-modified DNA. Here we present an alternative approach called SCL-exo which combines selective chemical labeling (SCL) of 5hmC in genomic DNA with exonuclease (exo) digestion of the bead-trapped modified DNA molecules. Associated with a straightforward bioinformatic analysis, this new procedure provides an unbiased and fast method for mapping this epigenetic mark at high resolution. Implemented on mouse genomic DNA from *in vitro*-differentiated neural precursor cells, SCL-exo sheds light on an intrinsic lack of conservation of hydroxymethylated CpGs across vertebrates.

**Electronic supplementary material:**

The online version of this article (doi:10.1186/s13059-016-0919-y) contains supplementary material, which is available to authorized users.

## Background

The recently discovered epigenetic mark 5-hydroxymethylcytosine (5hmC) results from an active DNA demethylation process which involves iterative oxidation of 5-methylcytosine (5mC) driven by Ten-eleven translocation (TET) enzymes and leads to the replacement of 5mC by an unmodified base [1–4]. However, 5hmC is stable enough to be readily detected in DNA, suggesting that, in addition to being an intermediate of DNA demethylation, it may have signaling potential by itself [5]. Hence, genome-wide mapping studies are valuable to understand the function of 5hmC and its importance in gene regulation. Pioneer studies have shown that 5hmC is found almost exclusively (99.89 %) in a CpG dinucleotide context in embryonic stem (ES) cells and pointed to a positive role of 5mC oxidation to 5hmC in the regulation of transcriptional enhancers as well as gene expression [5–11]. Indeed, high 5hmC levels correlate with active chromatin features at enhancers (i.e. H3K4me1 and H3K27ac) and with expression levels in gene bodies. Depending on the expected resolution, several different strategies can be used to map 5hmC. Low resolution (200–300 bp) methods employ hydroxymethylated DNA capture, either with antibodies (hydroxymethylated DNA immunoprecipitation (hMeDIP)) [8] or with streptavidin beads after 5hmC glucosylation and biotinylation (selective chemical labeling (SCL)) [9]. Such methods are sufficient to describe the presence of 5hmC in short genomic regions but, since resolution depends on the size of the DNA fragments, do not allow a precise mapping of the modified base. Whenever single-base resolution is required, for instance to analyze 5hmC distribution with respect to transcription factor binding sites (TFBSs), two methods based on bisulfite (BS) modification of DNA can be used [11, 12]. The first one uses 5hmC protection by glucosylation coupled to 5mC oxidation by recombinant TET followed by BS modification and sequencing (TAB-seq) [11]. The second procedure requires a chemically-induced oxidative deprotection of 5hmC followed by BS modification and sequencing (oxBS-seq) [12]. In the latter, results need to be compared to data obtained with an unmofidied BS-seq procedure which does not discriminate 5mCs from 5hmCs [13]. Although often defined as gold standards, BS-seq-based methods suffer from several drawbacks: (1) efficiency of TAB-seq relies on the use of a highly active recombinant TET enzyme; (2) harsh oxBS conditions lead to a substantial loss of DNA (99.5 % [12]) and a fairly good correlation between two biological replicates was achieved only after pooling CpG hydroxymethylation scores in given CpG islands [12]; (3) the current elevated cost of a full genome coverage can be prohibitive; and (4) they require complex bioinformatics [14]. Alternatively, 5hmC can be mapped at single-base resolution through two rounds of M*spI* digestion of DNA separated by a 5hmC glucosylation step, before size selection and sequencing (RRHP) [15]. Although highly reproducible, this procedure does not cover all CpGs in the genome since M*spI* requires a CCGG context for DNA cleavage (i.e. 15 % of all CpGs). In addition, restriction enzymes from the P*vuRts1I* family like A*baSI* have been shown to cleave glucosylated 5hmC-containing DNA and to be suitable for genome-wide mapping of the modified base [16]. However, due to their specific sequence requirement and restriction characteristics, theoretically only 58 % of all cytosines can be covered [17], and ambiguity might exist in 13 % of the cleaved molecules in the Aba-seq assay [16].

In an effort to develop an alternative approach for single-CpG resolution mapping of 5hmC genome-wide, we adapted a strategy first employed to increase the resolution of chromatin immunoprecipitation (ChIP) through the use of an exonuclease (exo) to trim DNA cross-linked to proteins up to close vicinity of intermolecular bounds (ChIP-exo [18, 19]). This new procedure, called SCL-exo, is shown here to be suited to obtain single-CpG resolution data. Using this approach, we uncovered that, although being included in highly conserved regulatory regions of the mouse genome, a majority of hydroxymethylated cytosines are not conserved in other vertebrate species, suggesting that they might affect chromatin structure rather than directly regulate transcription factor binding.

## Results and discussion

**Fig. 1:**
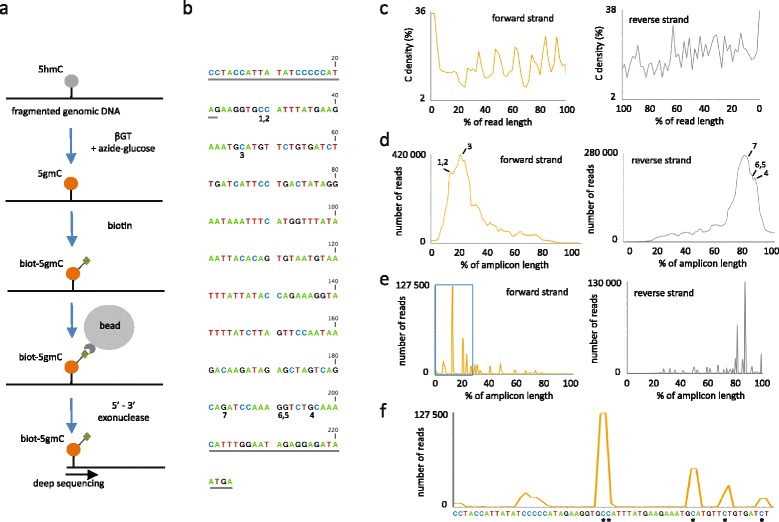
SCL-exo of a 5hmC-containing DNA standard. **a** Schematic representation of the SCL-exo procedure. Note that, for the sake of clarity, only single-stranded DNA is shown. **b** Sequence of the forward strand of a 224-bp hydroxymethylated DNA standard obtained by PCR amplification of mm8 chr3:93,697,590-93,697,813, using 5hmdCTP instead of dCTP. Sequences corresponding to the primers are underlined and do not contain 5hmCs. All other cytosines were hydroxymethylated. Positions of the three first 5hmCs of the forward strand and of the four first 5hmCs of the reverse strand have been numbered 1, 2, 3 and 4, 5, 6, 7, respectively. **c** Cytosine density along read length (10,000 reads for each strand). **d** Number of reads covering each position along the DNA standard for both forward and reverse strands. Numbering on the graph indicates the 5hmCs identified 1, 2, 3 and 4, 5, 6,7 in (**b**). **e** Coverage of each C of the DNA standard found within 10 bases from the start of all reads. **f** Close-up view of the signal shown within the *blue box* in (**e**) and associated to the first 60 bases of the forward strand. The sequence is shown below and 5hmCs have been marked by *asterisks*

Mouse epiblast-like P19 embryonal carcinoma cells were treated with retinoic acid (RA) for 48 h to induce their differentiation into neural progenitor-like cells (NPLCs) [5]. Genomic DNA was then fragmented by sonication and 5hmCs were glucosylated in vitro using β-glucosyltranferase and azide-glucose (5gmC, Fig. 1a). Azide then reacted with a biotin conjugate allowing immobilization of the modified DNA (biot-5gmC, Fig. 1a) on streptavidin-coated magnetic beads. After end-polishing and adapter ligation as previously described [19], captured DNA was then treated on beads with 5’-3’ exonuclease. After elution from the beads, samples were processed for subsequent library preparation and Illumina sequencing. Applying SCL-exo to a hydroxymethylated DNA standard (Fig. 1b) revealed that, as expected, a large fraction of sequencing reads started with a C (i.e. 36 % for the forward strand and 38 % for the reverse strand, Fig. 1c). In addition, the number of reads covering each base within the DNA standard peaked at the first hydroxymethylated Cs of both strands, indicating exonuclease stalling at bead-bound biot-5gmCs (Fig. 1d–f). It is of note that not all DNA strands were digested by the exonuclease up to the first 5hmC since unmodified Cs were found within reads (Fig. 1e and f). In addition, conversion of 5hmC to biot-5gmC is likely to be incomplete since the exonuclease did not stall systematically at the first modified C (a fraction of the reads were covering sequences located more than 40 bases away from the first hydroxymethylated cytosine of the standard, Fig. 1d and e). Analyzing the number of reads covering bases upstream (up to position 19) of the first hydroxymethylated cytosine (position 29) of the DNA standard suggested that the exonuclease did not digest efficiently the 5’ end of the standard in 12.38 % of the cases. Similarly, the rate of lack of exonuclease stalling, probably due to a lack of glycosylation/biotinylation and/or binding to beads, could be inferred from the number of reads starting after the first hydroxymethylated cytosine and was found to be 51.04 %. Accordingly, the probability of not identifying a 5hmC in a replicate of SCL-exo is: 0.1238 + 0.5104 = 0.6342. However, when addressing CpG hydroxymethylation, taking into account information from both strands leads to a probability of not identifying a 5hmCpG of 0.6342^2^ (0.4022). In the case of two replicates, the probability to identify a 5hmCpGs is thus (1–0.4022^2^) × 100 = 83.82 % and raises to 93.49 % when running three replicates. Hence, it is crucial to run several SCL-exo replicates in order to improve 5hmCpGs identification.

**Fig. 2:**
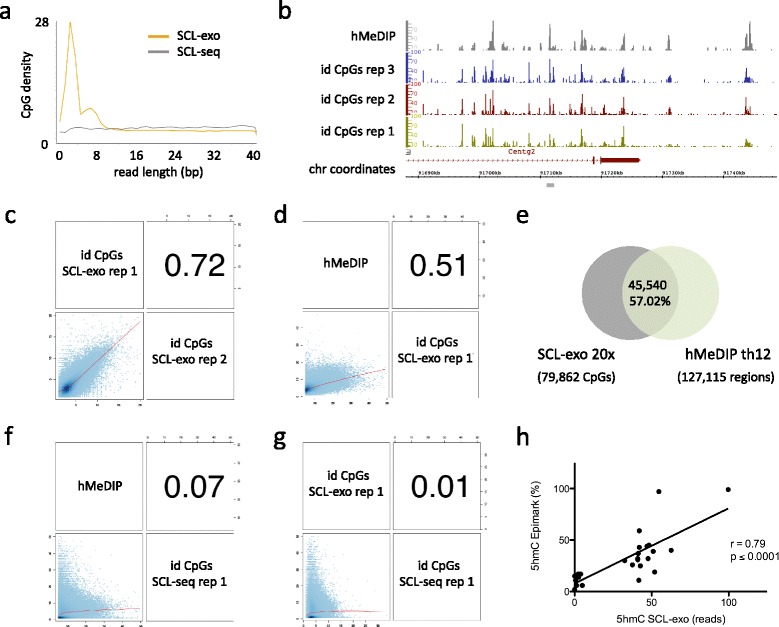
Validation of the SCL-exo strategy for single-CpG resolution mapping of 5hmC in genomic DNA from P19 cell-derived NPLCs. **a** Average CpG density along read length from SCL-seq (375,104 reads from chr7) and from SCL-exo (899,652 reads from chr7). **b** IGB visualization of hMeDIP and SCL-exo signals from three technical replicates in the 3’ region of the *Centg2* gene. **c** Genome-wide correlation coefficient value (Pearson’s coefficient, r) for.wig files corresponding to two technical replicates of SCL-exo identified (id) CpGs. **d** Genome-wide correlation coefficient value for one replicate of hMeDIP and one replicate of SCL-exo. **e** *Venn diagram* indicating the percentage of SCL-exo id CpGs (called from the consensus.wig file with a coverage threshold of 20×) included in hMeDIP-seq peaks (called with a threshold (*th*) of 12×). **f** Genome-wide correlation coefficient value for one replicate of hMeDIP and one replicate of SCL-seq. **g** Genome-wide correlation coefficient value for one replicate of SCL-exo and one replicate of SCL-seq. **h** Correlation between SCL-exo signal at id CpGs (number of reads) and their percentage of hydroxymethylation determined with the EpiMark kit for 27 selected CCGG sites (r: Pearson’s correlation coefficient)

In the context of hydroxymethylated CpGs in NPLC genomic DNA, the exonuclease stalled on average 3 bp, and in most cases within a window of 10 bp, upstream of bead-bound biot-5gmCs (Fig. 2a). Reads obtained by single-end HiSeq sequencing of three technical replicates were next mapped to the mouse reference genome and processed as follows to generate SCL-exo signal files: reads including a single CpG within a 10-bp window starting at the 5’ end (42 % of all mapped reads, Additional file 1a) were selected to build single-CpG resolution.wig files whose positions identify hydroxymethylated CpGs (id CpGs) and whose signal represents the coverage of that position (i.e. the sum of the reads from both strands at each given CpG). To avoid calling ambiguities, reads containing two or more CpGs in the first 10 bases (4.7 % of all mapped reads, “probable CpGs” in Additional file 1a) were not retained for analysis. Visualization by the Integrated Genome Browser (IGB) software showed that SCL-exo signal at id CpGs correlated well with hMeDIP-seq peaks (Fig. 2b). Similarly, reads containing CpHs and no CpGs in the first 10 bases (47 % of all mapped reads, Additional file 1a) were used to build a.wig file putatively reflecting non-CpG hydroxymethylation. IGB visualization indicated that the putative CpH hydroxymethylation signal was distributed quite uniformly with no detectable peaks corresponding to hMeDIP peaks (Additional file 1b). In addition, no correlation (Pearson’s correlation coefficient [r] = 0.04) was detected between possible CpH signals from two technical replicates (Additional file 1c). Collectively, these data strongly suggest that, as shown for mouse ES cells [11], most hydroxymethylated cytosines are found in a CpG context in RA-treated P19 cells and that the occurrence of SCL-exo reads not containing CpGs in their first 10 bases was probably due to a suboptimal processing by the exonuclease.

In order to evaluate the reproducibility of the SCL-exo procedure Pearson’s correlation coefficient was determined for id CpG.wig files from two technical replicates of SCL-exo (Fig. 2c). Signals from SCL-exo id CpG replicates showed a high correlation (*r* = 0.72), indicating that SCL-exo is suited for a reproducible identification of hydroxymethylated CpGs. Notably, non-overlapping SCL-exo id CpGs between two replicates had a lower coverage than overlapping id CpGs (Additional file 1d). Hence, increasing sequencing depth might enhance the reproducibility of the method. Considering that the mean signal of overlapping id CpGs was 1.6-fold higher than the mean signal of non-overlapping id CpGs from two replicates with 48 million reads, increasing sequencing depth up to 1.6 × 48 million reads (≈80 million reads) per replicate could allow higher confidence in the identification of hydroxymethylated CpGs. Finally, SCL-exo id CpG signal showed a fairly good correlation with hMeDIP (*r* = 0.51, Fig. 2d) and 57.02 % of the SCL-exo id CpGs with at least 20× coverage were included in hMeDIP peaks (Fig. 2e). As a possible readout of exonuclase undigested DNA fragments, we selected unique CpGs contained in the first 10 bases of reads obtained by SCL-seq without exonuclease digestion to build a SCL-seq id CpG.wig file. This SCL-seq id CpG signal did not correlate with hMeDIP (*r* = 0.07, Fig. 2f) and SCL-exo (*r* = 0.01, Fig. 2g). The mean signal (number of reads) at SCL-seq id CpGs was 2.03 and could thus be considered as a threshold for false identification of hydroxymethylated CpGs. Hence, for the subsequent analysis, only CpGs identified in at least two out of three SCL-exo replicates (consensus id CpGs) at a threshold arbitrarily set to 8× coverage were considered (178,218 id CpGs). The status of hydroxymethylation of 27 selected CpGs from this set (consensus id CpGs) and included in a M*spI* CCGG restriction site was next verified by using the EpiMark 5-hmC and 5-mC Analysis Kit (New England Biolabs) which allows a quantitative determination of the percentage of hydroxymethylation of CpGs thanks to the insensitivity of glucosylated ChmCGG sites to M*spI* cleavage. A strong correlation (*r* = 0.79) between EpiMark and SCL-exo data was observed (Fig. 2h and Additional file 1e). This is in the range of what has been observed when Aba-seq and EpiMark data were compared (*r* = 0.72) for hydroxymethylated CpGs in ES cells [16]. Interestingly, the hydroxymethylated status of SCL-exo id CpGs not included in hMeDIP peaks was systematically validated with the EpiMark kit, thus indicating that those were not false positive (Additional file 1e). Comparison between the two techniques suggested however that below a threshold of 8 % of hydroxymethylation (as assessed by EpiMark), CpGs were not efficiently identified by SCL-exo (Fig. 2h and Additional file 1e). Here again, increasing sequencing depth might increase the identification rate of these poorly hydroxymethylated CpGs. This correlation study allowed us to estimate that CpGs showing 20 % hydroxymethylation by EpiMark should have an approximate 16× coverage by SCL-exo. Using this threshold of coverage, the calculated overlap between id CpGs from two replicates of SCL-exo was 53.6 %. The genome-wide distribution of SCL-exo id CpGs and probable CpGs was next interrogated with the CEAS annotation tool [20]. As already described for 5hmC-enriched regions from P19 cells recovered by immunoprecipitation [5], SCL-exo id CpGs were particularly enriched in introns (*p* = 9.7e^−256^) and promoters (*p* = 1.1e^−49^), although exons might be slightly under-represented due to the fact that “probable CpGs,” which are found in exons for 6.7 % of them, were not included in the analysis (Additional file 1f). In addition, inclusion of id CpGs in enhancers (H3K4me1 positive regions), either active (positive for H3K27ac) or primed (negative for H3K27ac), was proportional to the depth of coverage, suggesting that SCL-exo-identified CpGs with high coverage are likely to be included in functional enhancers.

**Fig. 3:**
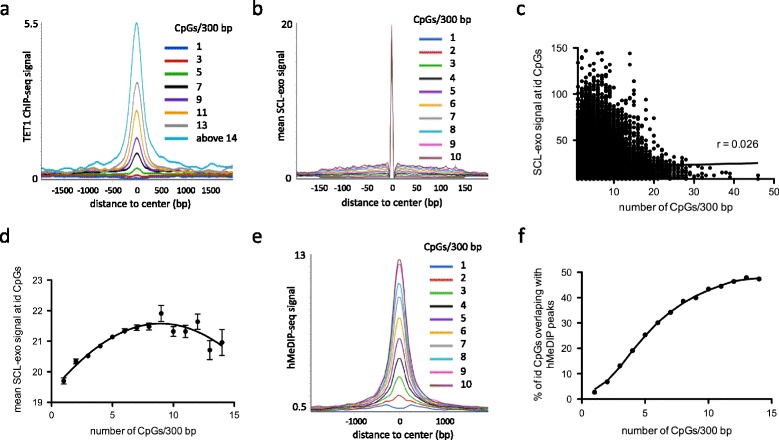
5hmC detection by SCL-exo in genomic DNA from NPLCs does not depend on CpG density. **a** Average TET1 ChIP-seq signal in 4000 bp regions around SCL-exo id CpGs sorted according to their CpG density (number of CpGs per 300 bp). **b** Mean SCL-exo signal at id CpGs sorted according to their surrounding CpG density. **c** *Scatter plot* of the SCL-exo signal at id CpGs as a function of CpG density (r: Pearson’s correlation coefficient). **d** Mean SCL-exo signal at SCL-exo id CpG as a function of CpGs density. **e** Mean hMeDIP-seq signal at id CpGs sorted according to their surrounding CpG density. **f** Inclusion of SCL-exo id CpGs in hMeDIP peaks as a function of CpG density

Since SCL-exo identified hydroxymethylated CpGs in genomic regions not enriched by immunoprecipitation (hMeDIP), a technique which efficacy is known to depend on CpG density [10], the relationship between CpG abundance in regions containing SCL-exo id CpGs and the associated SCL-exo coverage was next investigated and compared to hMeDIP and TET1 ChIP-seq signals from RA-treated P19 cells (GSM941665 and GSM941681 respectively). Although TET1 enrichment was strongly correlated to CpG density (Fig. 3a), such a correlation was not observed for the SCL-exo signal at id CpGs (Fig. 3b–d). Indeed, id CpGs in regions with a unique CpG in 300 bp had a fairly high mean coverage (19.70 ± 0.1), the maximum mean id CpG coverage (reached for regions with 9 CpGs in 300 bp) being only 1.11-fold higher (21.91 ± 0.26 - Fig. 3d). Conversely, hMeDIP-seq signal was clearly dependent on CpG density and regions with a unique hydroxymethylated CpG were not immunoprecipitated (Fig. 3e). Accordingly, inclusion of SCL-exo id CpGs in hMeDIP peaks increased as a function of CpG density (Fig. 3f). These data indicate that SCL-exo is more sensitive than hMeDIP for detecting hydroxymethylated CpGs at low density.

**Fig. 4:**
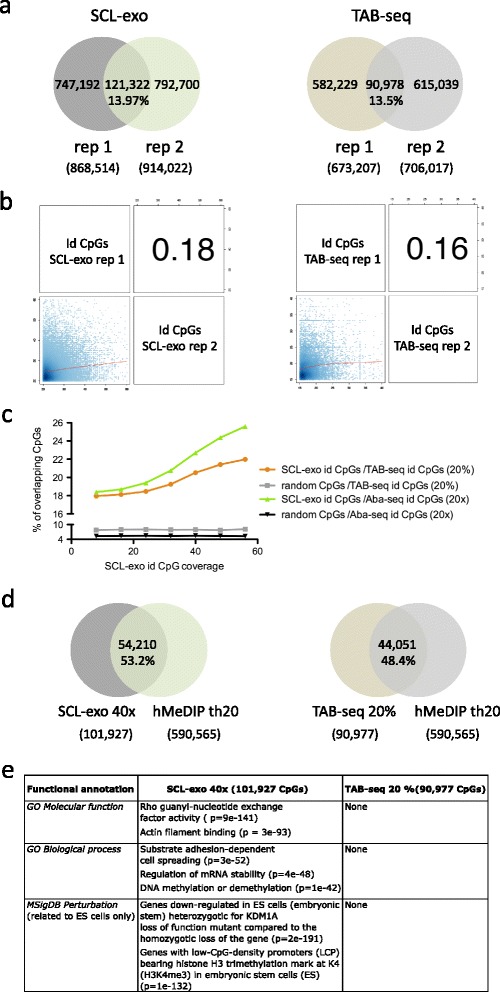
5hmCpG mapping by SCL-exo in mouse ES cells. **a** *Venn diagrams* indicating the percentage of overlapping id CpGs between two technical replicates of SCL-exo (*left diagram*) and two technical replicates of TAB-seq (*right diagram*) in E14 mESCs. SCL-exo id CpGs were selected for having a coverage ≥ 20× and TAB-seq id CpGs for being at least 20 % hydroxymethylated. **b** Genome-wide correlation coefficient value for two technical replicates of SCL-exo (*left panel*) and TAB-seq (*right panel*). Signals were compared for id CpGs with between 20× and 60× coverage in SCL-exo and with between 15 % and 40 % of hydroxymethylation in TAB-seq. **c** Graph representing the percentage of overlapping CpGs between either SCL-exo and TAB-seq (hydroxymethylation ≥ 20 %) or SCL-exo and Aba-seq (coverage ≥ 20×, 2,320,973 CpGs), as a function of the coverage of SCL-exo id CpGs. For each SCL-exo coverage value a similar number of CpGs were randomly picked among the 21,342,492 CpGs of the mm9 genome, and submitted to the same analysis. **d** *Venn diagrams* indicating the percentage of id CpGs from either SCL-exo (called from the consensus.wig file with a threshold (*th*) of 40×, *left diagram*) or TAB-seq (hyroxymethylation ≥ 20 %, *right diagram*), overlapping with hMeDIP-seq peaks (called with a threshold of 20×). **e** Functional annotation of the SCL-exo and TAB-seq identified 5hmCpGs. Annotation was done with GREAT (http://bejerano.stanford.edu/great/public/html/) and binomial raw *p* values are given in brackets for each item

We next implemented SCL-exo with genomic DNA from E14 mouse ES cells (see sequencing statistics in Additional file 2a) and compared the data with previously published TAB-seq or Aba-seq datasets in E14 ES cells [16, 21]. Surprisingly, hydroxymethylated CpGs were not consistently identified within the three technical replicates of SCL-exo (Additional file 2b) and only 13.97 % of id CpGs with at least 20× coverage overlapped between two replicates (Fig. 4a), whereas their SCL-exo signals showed a rather poor correlation (*r* = 0.18, Fig. 4b). Most notably, a similar lack of reproducibility was observed for high confidence (≥20 % hydroxymethylation) CpGs identified by TAB-seq (Fig. 4a and b, right panels). However, running the SCL-exo CpG identification algorithm on the E14 input-seq reads showed that the number of false positive CpGs identified within the Input-seq dropped abruptly when coverage increased (Additional file 2c) whereas the number of SCL-exo id CpGs remained quite stable within the same range of coverage, indicating that SCL-exo id CpGs in E14 cells are most likely to be truely hydroxymethylated. Collectively, these data suggest that the E14 mESC hydroxymethylome might be extremely variable from cell to cell, a conclusion which is in accordance with the known variability of the methylome of ES cells [22–24]. Despite this variability, a significant fraction of the CpGs identified either by SCL-exo or by TAB-seq overlapped, especially at high coverage (Fig. 4c). This was also observed when comparing SCL-exo and Aba-seq data (Fig. 4c). Importantly, as noticed for RA-treated P19 cells, a large fraction of SCL-exo id CpGs fell within hMeDIP-seq peaks (Fig. 4d, left panel). This was also true for TAB-seq id CpGs (Fig. 4d, right panel), but variability was still observed between TAB-seq and SCL-exo id CpGs overlapping with hMeDIP peaks (Additional file 2d). These data indicate that, although cell to cell heterogeneity in E14 ESCs might hinder reproducible identification of unique 5hmCpGs, SCL-exo and TAB-seq identify similar regions as being hydroxymethylated in E14 ESCs. Accordingly, functional annotation of the SCL-exo and TAB-seq id CpGs overlapping hMeDIP peaks in E14 cells using GREAT (http://bejerano.stanford.edu/great/) generated similar terms for both sets of CpGs (Additional file 2e). However, analysis of the bulk id CpGs from both techniques (i.e. without selecting those overlapping with hMeDIP peaks) using standard settings of the GREAT annotation tool retrieved functional annotation terms only for SCL-exo id CpGs (Fig. 4e).

**Fig. 5:**
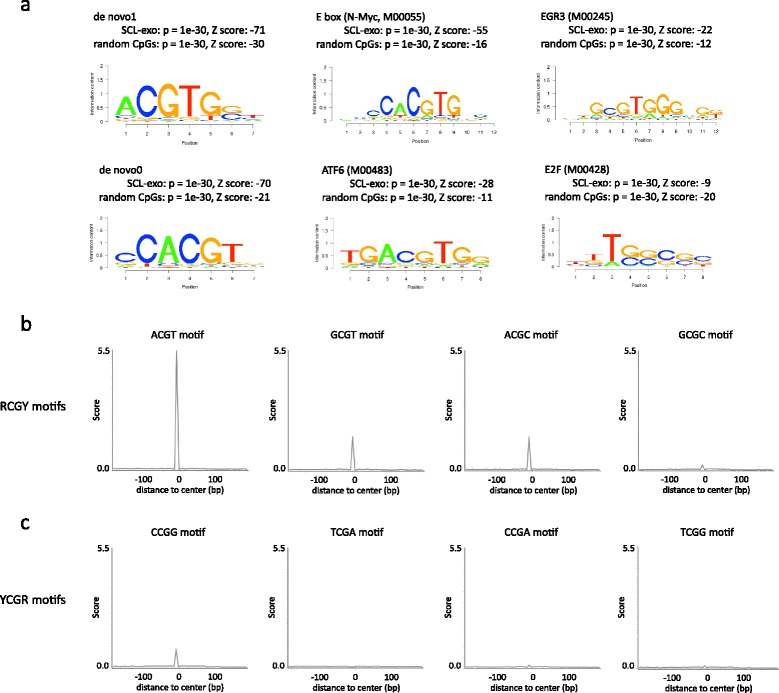
Association of 5hmCs with DNA motifs. **a** Logos of de novo motifs and transcription factor binding motifs retrieved by the *SeqPos* motif tool from *Cistrome* (http://cistrome.dfci.harvard.edu) in 100 bp sequences centered on SCL-exo id CpGs included in hMeDIP peaks. As a control, motif search was run on 100 bp sequences centered on randomly selected CpGs. For each logo, the associated *p* value and z-score are indicated. **b**, **c** CpG hydroxymethylation prefers RCGY to YCGR motifs. Average profiles of RCGY (**b**) and YCGR (**c**) motif densities (R = A or G, and Y = C or T) around 27,031 SCL-exo id CpGs with at least 30× coverage

Aside from generating information on the location of 5hmC-enriched regions in the genome, single-CpG resolution of SCL-exo allows to interrogate databases for particular TFBS motif enrichment with high precision. To this aim, sequences including hydroxymethylated CpG positions in P19-derived NPLCs were searched for motifs with the *SeqPos* motif tool from *Cistrome* (http://cistrome.dfci.harvard.edu, [25]). Retrieved motifs included the CpG-containing E box (N-Myc), E2F, ATF6, and EGR motifs with the highest probability (*p* = 1e^−30^, Fig. 5a). These particular motifs were also found to be enriched (*p* = 1e^−30^) in a set of CpG-containing sequences picked at random in the genome (Fig. 5a). Nonetheless, Z-scores calculated by *SeqPos* clearly indicated a specific enrichment for the CGTG-containing E-box, ATF, and EGR motifs in SCL-exo identified regions versus random CpG regions (Fig. 5a). These data suggest that TET targeting is biased towards CpGs included in a CGTG motif and, as a consequence, that DNA methylation/demethylation could regulate the activity of E-box, EGR and ATF motif-containing regions on a wide scale in vivo, as already suggested for unique regions [26–28]. However, according to their Z-score, the de novo motifs ACGTG and CACGT ranked before known TFBSs (Fig. 5a). These two motifs shared the ACGT sequence which was previously shown to be preferentially methylated by the DNA methyltransferases 3a and 3b (DNMT3s) together with other RCGY (R = A or G, and Y = C or T) motifs compared to YCGR motifs [29]. Accordingly, a higher incidence of CpG hydroxymethylation at RCGY motifs compared to YCGR motifs was evidenced through the analysis of motif densities around SCL-exo id CpGs (Fig. 5b and c). Since TETs might not have sequence selectivity as suggested by the structure of TET2 complexed with DNA [30], the detected bias in TET targeting towards RCGY motifs might actually reflect a preferential targeting of these motifs by DNMT3s in P19 cells.

**Fig. 6:**
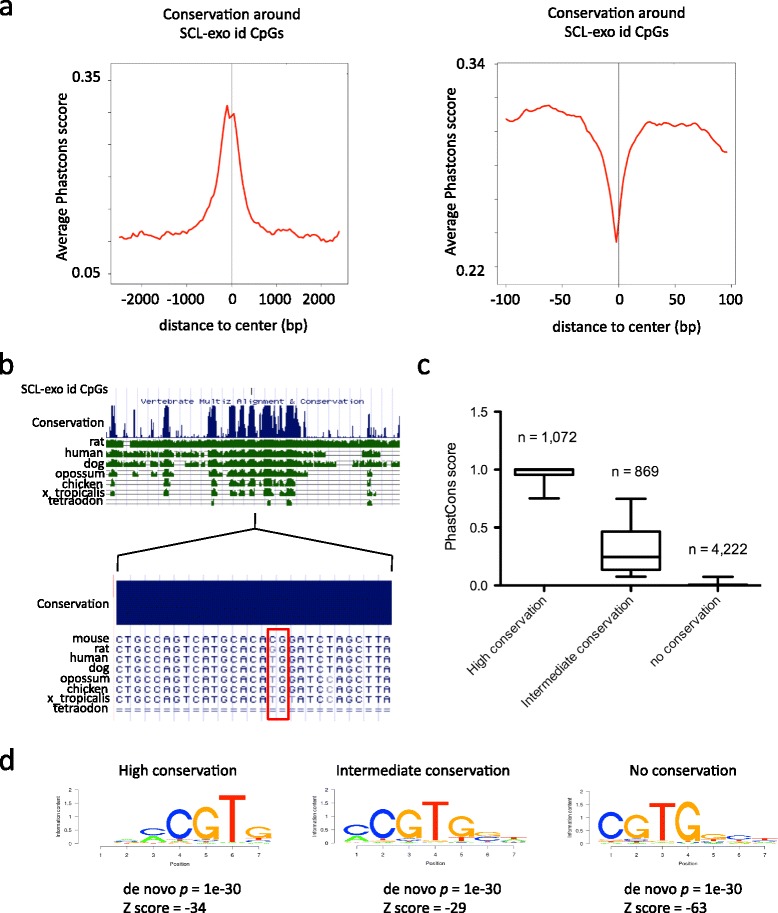
SCL-exo id CpGs show low conservation among vertebrates. **a** Average PhastCons score around SCL-exo id CpGs found within a Meis1 ChIP-seq peak either in a 5000 bp window (*left panel*) or in a close-up view of 200 bp (*right panel*). **b** Screenshot of UCSC genome browser showing the conservation between species of sequences including a SCL-exo id CpG (*boxed in red*). **c** SCL-exo id CpGs were sorted into three groups: High conservation (PhastCons score between 0.75 and 1), Intermediate conservation (PhastCons score between 0.075 and 0.75), and No conservation (PhastCons score between 0 and 0.075). *Box plots* illustrate the distribution of the PhastCons score for the three groups of id CpGs. **d** Best ranked motif for each of the three groups of id CpGs sorted according to their conservation, as determined by the *SeqPos* motif tool from *Cistrome*

Cytosine hydroxymethylation has been associated with the activity of enhancers, as evidenced for those bound by the homeodomain transcription factor Meis1 [5]. Enhancers are genomic regions enriched in multiple TFBSs which are characterized by their conservation across species [31]. In order to further investigate a potential relationship between hydroxymethylated CpGs and transcription factor binding, the conservation status of SCL-exo id CpGs from a subset of enhancers encompassing ChIP-seq-identified Meis1-bound TGACAG binding sites in NPLCs was examined. Although id CpGs were included in highly conserved genomic regions (Fig. 6a, left panel), these CpGs were themselves poorly conserved (Fig. 6a, close-up view, right panel). As an example, Fig. 6b shows that, although embedded in a highly conserved region, the observed SCL-exo id CpG is present only in the mouse genome. The lack of conservation of hydroxymethylated CpGs could also be inferred from TAB-seq data in E14 ES cells (Additional file 3a), indicating that it is not an artifact due to the SCL-exo method and, most importantly, that it is also true for ES cells. Conversely, Meis1-bound TGACAG motifs in P19 cells showed high conservation, as did c-Myc- and N-Myc-bound CACGTG motifs in ES cells, whereas SCL-exo id CpGs overlapping with CACGTG sites showed a lack of conservation (Additional file 3b–e). These data indicate that hydroxymethylated CpGs are not conserved among vertebrates and highlight the possibility that transcription factor-bound CpGs are protected from loss during evolution by a lack or a high turnover of cytosine modifications. In support of this hypothesis, c-Myc- and N-Myc-bound CACGTG motifs in ES cells were not found to be enriched in 5hmC by TAB-seq (Additional file 3f), and only 14 N-Myc-bound motifs (out of 313) were identified by SCL-exo (Additional file 3g). Of note, these 14 motifs showed a drop in conservation at the level of the CpG dinucleotide (Additional file 3h). Next, SCL-exo id CpGs from NPLCs were clustered into three groups according to their level of conservation (Fig. 6c). As expected, a majority of id CpGs showed extremely low to no conservation (i.e. 68.5 % had a PhastCons score below 0.075). These clusters were analyzed for motif enrichment (Fig. 6d), genomic distribution (Additional file 3i), SCL-exo signal (Additional file 3j), and H3K4 monomethylation (Additional file 3k), and results showed that regions containing hydroxymethylated CpGs share similar characteristics, independently of their CpG conservation status. Hence, these results suggest that conservation during evolution does not represent a driving force for CpG targeting by TETs.

## Conclusions

Collectively, our data indicate that 5hmC can be mapped at single-CpG resolution by SLC-exo. Contrary to methods based on the use of restriction enzymes, SCL-exo has the advantage of being unbiased in terms of sequence context requirement. Using SCL-exo, we demonstrate here that TET enzymes mainly target unconserved CpGs, suggesting that cytosine hydroxymethylation at enhancers might serve a structural role at the level of chromatin rather than having a direct effect on transcription factor binding to DNA.

## Methods

### Cell culture and genomic DNA preparation

P19.6 embryonal carcinoma cells were culture as described [5] in high glucose Dulbecco’s Modified Eagle Medium supplemented with 10 % fetal calf serum (GIBCO, USA). Neural progenitor cell differentiation was triggered by 10^−6^ M *all-trans* retinoic acid (RA) in 10-cm diameter culture dishes. Cells were scraped in phosphate buffered saline 48 h after RA addition and were pelleted at 100 g before genomic DNA extraction using a DNeasy Blood and Tissue kit (Qiagen, France). Mouse ES cells (E14) were grown in high glucose Dulbecco’s Modified Eagle Medium supplemented with 15 % fetal calf serum, 0.1 mM β-mercaptoethanol, 1X non-essential amino acids, and LIF (1000 U/mL). Genomic DNA from E14 cells was extracted with the DNeasy Blood and Tissue kit (Qiagen).

### SCL-seq and SCL-exo procedures

A total of 20 μg of DNA in 300 μL of TE buffer (Tris 10 mM, EDTA 0.1 mM, pH 8.0) were sonicated with a Bioruptor (Diagenode, Belgium) to yield 200–500 bp fragments. Each glucosylation and biotinylation reaction was run using reagents from the Hydroxymethyl Collector kit (ref. 55013, Active Motif, Belgium) and 500 ng of sonicated DNA. For SCL-seq, three technical replicates each with 2.5 μg biotinylated genomic DNA were captured on streptavidin-coated magnetic beads (ref. 11205D, Invitrogen). After five washes and elution from the beads according to the manufacturer’s protocol, the captured DNA was purified, precipitated, and pooled for sequencing library preparation using the TruSeq ChIP Sample Prep Kit (Illumina, ref. IP-202-1012). For SCL-exo, three technical replicates each which 2.5 μg of biotinylated genomic DNA were captured on streptavidin-coated magnetic beads. After six washes in RIPA buffer (50 mM HEPES pH 7.6; 1 mM EDTA; 0.7 % Na-Deoxycholate; 1 % NP-40; 0.5 M LiCl) and two washes in Tris 10 mM pH 8, the DNA-beads complexes were processed as previously described [19]: end polishing, ligation of the P7 exo-adapter, nick repair, *lambda* and R*ecJ*f exonuclease digestion, elution, P7 primer extension, ligation of the P5 exo-adapter, PCR amplification, and finally gel-size selection. The exonuclease-digested DNA was eluted from the beads by incubation in 100 μL of Elution Buffer (95 % formamide, 10 mM EDTA) at 90 °C for 5 min, followed by DNA precipitation and resuspension in 20 μL of water. The SCL-seq and SCL-exo libraries were quantified using the KAPA library quantification kit for Illumina sequencing platforms (KAPA Biosystems, KK4824) and 50 bp single-end sequenced as a pool in a single lane of a HiSeq 2000 (Illumina) for RA-treated P19 cells or in four lanes of a HiSeq 2500 (Illumina) for E14 mESCs, following the manufacturer’s protocol. Sequencing data are available at the NCBI GEO database under reference GSE70635.

### ChIP-seq procedure

After 48 h of all-trans retinoic acid treatment, P19.6 cells were cross-linked in 10 mL PBS 1 % formaldehyde for 10 min at room temperature. The reaction was stopped by adding 1 mL of 1 M Glycine. Cells were washed twice in cold PBS, scrapped, and pelleted at 100 g. The ChIPs were performed as described previously [32] with chromatin from 50.10^6^ cells, using 10 μg of anti-H3K4me1 (Abcam, ref. ab8895) and anti-H3K27ac (Abcam, ref. ab4729) antibodies. The ChIP-seq libraries were prepared using the TruSeq ChIP Sample Prep Kit (Illumina, ref. IP-202-1012) and sequenced on HiSeq 2000. Mapping to the mouse mm8 genome and peak calling were run as described previously [5].

### SCL-exo bioinformatics

SCL-exo fastq files were filtered using *SolexaQA* [33] to retain high-quality reads only (*Q* = 20, *l* = 17) before being mapped to mm8 (P19 cells) or mm9 (E14 cells), forward and reverse strands separately, using *Bowtie* [34] with parameters *l = 32 bp*, *n = 1*, *m = 1*, *strata*, *best*, and *Samtools* [35]. The bam files were then processed to generate.wig files using MACS 1.4.0 [36]. Resulting.wig files were filtered to remove UCSC blacklisted regions as well as few regions showing a very high signal and not included in the blacklists. Reads for which a single CpG was found within 10 bases from their 5’ end were selected to build a single-CpG resolution.wig file in which signal at id CpGs corresponds to the sum of the reads covering both Cs (two strands) at each given CpG. High confidence SCL-exo id CpGs (178,218 CpGs) were called when covered more than 8× and found in at least two out of three replicates. For the analysis of the reproducibility of SCL-exo identification of CpGs as a fonction of read density (Additional file 1d), 5000 CpGs identified in two replicates and 5000 CpGs identified in only one replicate out of two were randomly selected. TFBS motif search was run with the *SeqPos* motif tool from *Cistrome* [25] (which does not accept datasets with more than 5000 regions) within 100 bp windows centered either on 3682 id CpGs with a SCL-exo signal above 45 reads and overlapping with a hMeDIP peak or on 3682 randomly selected CpGs (Fig. 5). A *SeqPos* search for motifs according to the conservation of SCL-exo id CpGs was run on a pool of 6163 id CpGs from mouse chr11, covered at least 20×, and sorted into three groups according to their PhastCons scores: High conservation (1072 id CpGs with a PhastCons score between 0.75 and 1), Intermediate conservation (869 id CpGs with a PhastCons score between 0.075 and 0.75), and No conservation (4222 id CpGs with a PhastCons score between 0 and 0.075). Analysis of the conservation of CpGs included in Meis1-bound enhancers was run on 4959 high confidence SCL-exo id CpGs with more than 30× coverage and found within a Meis1 ChIP-seq peak (Fig. 6). Conservation of TAB-seq id CpGs was analyzed for 90,977 CpGs showing more than 20 % of hydroxymethylation in two replicates of TAB-seq (Additional file 3). In addition, 3887 Meis1-bound TGACAG motifs from RA-treated P19 cells were identified and assigned PhastCons scores. Similarly, 152 c-Myc bound CACGTG sites and 313 N-Myc bound CACGTG sites in mouse ES cells were included in this analysis. Finally, 245 CACGTG sites from RA-treated P19 cells and overlapping with SCL-exo id CpGs with more than 30× coverage were selected. All conservation graphs were generated with *Cistrome*.

### Datasets used in this study

TET1 ChIP-seq and hMeDIP-seq data from RA-treated P19 cells were from the Gene Expression Omnibus repository (GEO - http://www.ncbi.nlm.nih.gov/geo) datasets GSM941681 and GSM941665, respectively. MEIS1 chip-seq data from RA-treated P19 cells were from GSM819083. Mouse ESC CpG hydroxymethylation data were extracted from datasets of two technical replicates of TAB-seq (GSM1180306 and GSM1180307) and another biological replicate (GSM118308). Aba-seq and hMeDIP-seq data from E14 mouse ESCs were from GSE42898 and GSM1087009, respectively. N-Myc and c-Myc bound CACGTG sites in mouse ES cells were extracted from GSM288356 and GSM288356 GEO datasets, respectively.

### Availability of supporting data

The datasets supporting the results of this article (SCL-exo, Input-seq, SCL-seq, H3K4me1, and H3K27ac ChIP-seq) are available in the GEO repository under accession number GSE70635.

## Additional files

Additional file 1: Is a figure including sequencing statistics and additional information regarding the validation of the SCL-exo protocol and complementing Fig. 1. (PDF 609 kb) Additional file 2: Is a figure indicating SCL-exo statistics and analysis for E14 mESCs, and complements Fig. 4. (PDF 232 kb) Additional file 3: Is a figure including additional information on the conservation status of hydroxymethylated CpG and their genome wide distribution and inclusion in H3K4me1-marked regions. Additional file 3 complements Fig. 6. (PDF 500 kb)
