# Supplementary material for: Single-CpG resolution mapping of 5-hydroxymethylcytosine by chemical labeling and exonuclease digestion identifies evolutionarily unconserved CpGs as TET targets
Source: Genome Biol. 2016 Mar 29;17:56. doi: 10.1186/s13059-016-0919-y (PMC4810514; doi:10.1186/s13059-016-0919-y)
Supplement: Additional file 1: — Is a figure including sequencing statistics and additional information regarding the validation of the SCL-exo protocol and complementing Fig. 1. (PDF 609 kb) [file 13059_2016_919_MOESM1_ESM.pdf]

a

SCL-exo sequencing statistics:	rep1	rep2	rep3
number of reads Q20:	48 473 464	48 604 839	51 613 442
mapped reads:	81.5 %	82.2 %	82.8 %
forward strand:	19 751 252	19 969 978	21 379 321
reverse strand:	19 739 819	19 969 392	21 379 171
total:	39 491 071	39 939 370	42 752 492
number of reads id CpGs:	16 845 901 (42.65 %)	17 074 911 (42.75%)	18 145 732 (42.44 %)
number of reads probable CpGs:	1 892 595 (4.7 %)	1 921 493 (4.8 %)	2 230 440 (5.2 %)
number of reads possible CpH:	18 434 830 (46.68 %)	18 636 379 (46.66 %)	20 079 240 (46.96 %)

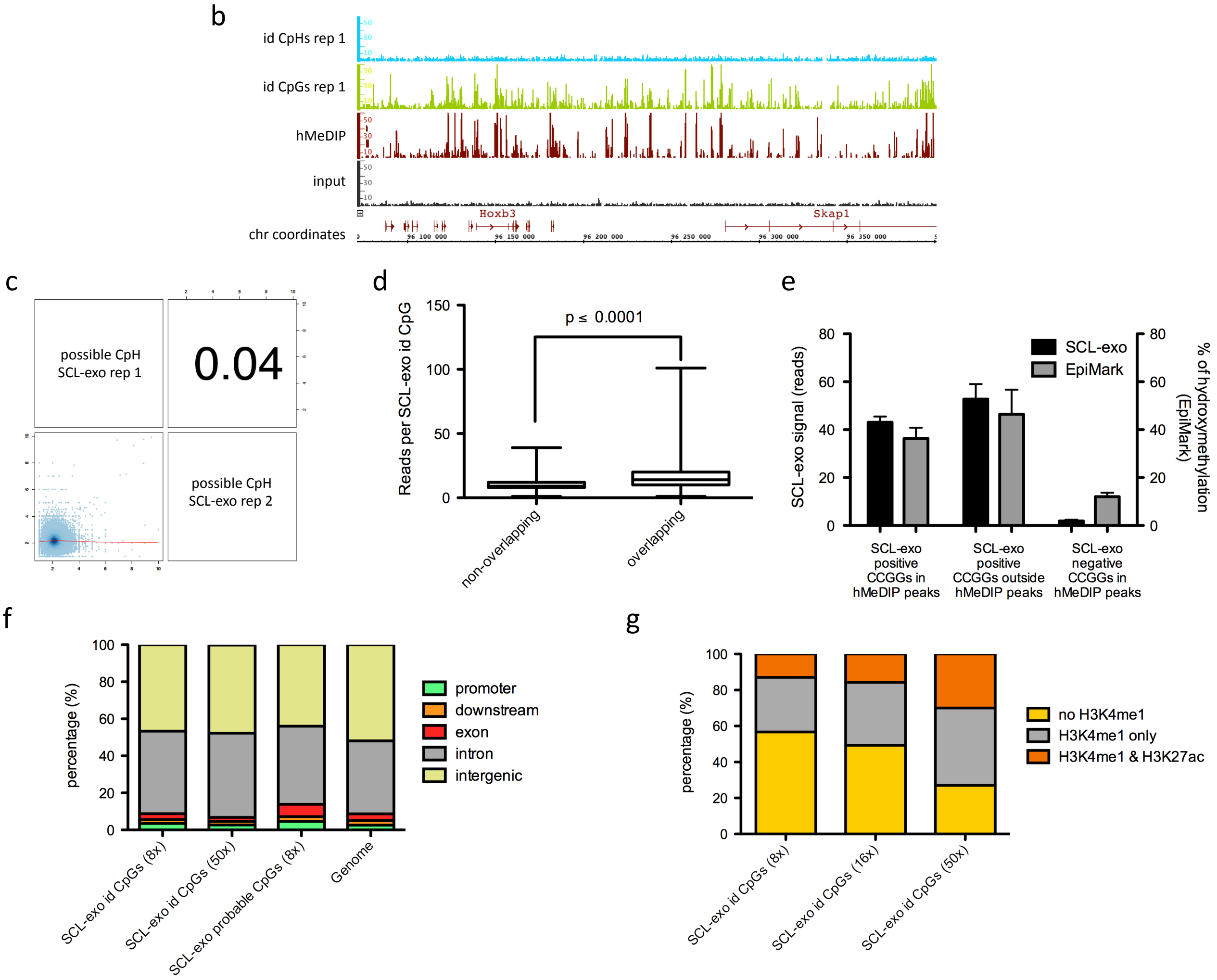

Additional file 1: Validation of the SCL-exo procedure in P19 cells. (**a**) Sequencing statistics for the three technical replicates of SCL-exo. SCL-exo id CpGs indicate reads with a single CpG within 10 bases from their start. Probable CpGs indicate reads with at least two CpGs within 10 bases from their start. Possible CpHs indicate reads with at least one C not followed by a G within 10 bases from their start. (**b**) IGB visualization of hMeDIP and SCL-exo signals at id CpGs or possible CpHs from technical replicate 1. (**c**) Genome wide correlation coefficient value (Pearson's coefficient, r) for possible CpH wig files from two technical replicates of SCL-exo. (**d**) Box plot analysis of the number of reads covering SCL-exo id CpGs from replicate 1 and overlapping or not with id CpGs from replicate 2. *P* was calculated by a Mann Whitney test with the GraphPad Prism software. (**e**) Average signal obtained by SCL-exo (number of reads) or EpiMark (percentage of hydroxymethylation) analysis of CCGG sites identified as (*i*) SCL-exo positive and found within a hMeDIP peak (n = 8), (*ii*) SCL-exo positive and not associated with a hMeDIP peak (n = 9) or (*iii*) SCL-exo negative and found within a hMeDIP peak (n = 10). (**f**) Gene-centered annotation of SCL-exo id CpGs with at least 8x or 50x coverage and of SCL-exo id probable CpGs with at least 8x coverage. Promoters are defined as -3000 bp to TSS and dowstream regions as TTS to +3000 bp. (**g**) Overlap between H3K4me1 and H3K27ac histone marks and SCL-exo id CpGs (RA-treated P19 cells) with at least 8, 16 or 50 fold coverage (8x, 16x and 50x respectively).
