# Supplementary material for: Single-CpG resolution mapping of 5-hydroxymethylcytosine by chemical labeling and exonuclease digestion identifies evolutionarily unconserved CpGs as TET targets
Source: Genome Biol. 2016 Mar 29;17:56. doi: 10.1186/s13059-016-0919-y (PMC4810514; doi:10.1186/s13059-016-0919-y)
Supplement: Additional file 2: — Is a figure indicating SCL-exo statistics and analysis for E14 mESCs, and complements Fig. 4. (PDF 232 kb) [file 13059_2016_919_MOESM2_ESM.pdf]

a

SCL-exo sequencing statistics:	rep1	rep2	rep3
number of reads Q20:	147 704 877	140 618 673	145 068 317
mapped reads:	78.1 %	77.1 %	77.3 %
forward strand:	57 658 724	54 214 971	56 040 735
reverse strand:	57 724 754	54 216 829	56 138 675
total:	115 383 478	108 431 800	112 179 410
number of reads id CpGs:	42 801 736 (37.1 %)	37 688 861 (34.8 %)	39 581 777 (35.3 %)
number of reads probable CpGs:	6 060 394 (5.3 %)	5 097 573 (4.7 %)	5 322 680 (4.7 %)
number of reads possible CpH:	58 840 341 (51 %)	57 720 134 (53.2 %)	59 359 150 (52.9 %)

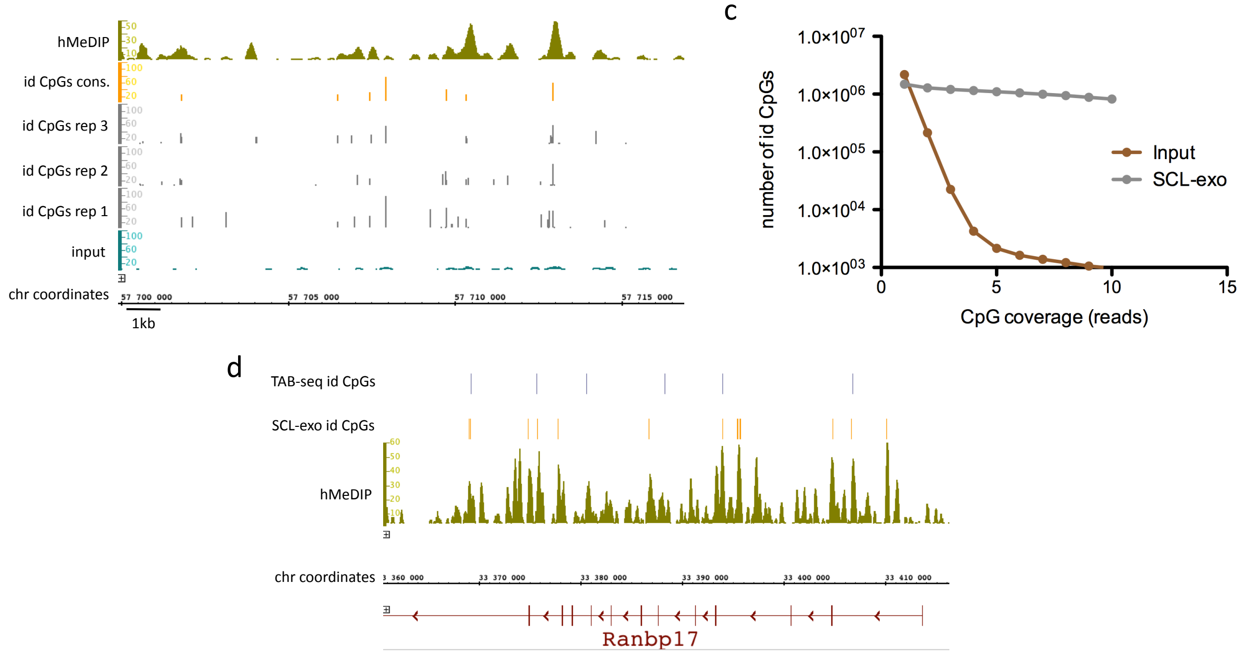

e

Functional annotation	SCL-exo 40x overlapping hMeDIP peaks (54,216 CpGs)	TAB-seq 20 %overlapping hMeDIP peaks (44,051 CpGs)
GO Molecular function	Rho guanyl-nucleotide exchange factor activity (p=2e-133)<br><br>Ras guanyl-nucleotide exchange factor activity (p=3e-130)<br><br>Actin filament binding (p = 3e-70)	Ras guanyl-nucleotide exchange factor activity (p=6e-92)<br><br>Rho guanyl-nucleotide exchange factor activity (p=2e-79)<br><br>Actin filament binding (p = 5e-52)
GO Biological process	Mesoderm morphogenesis (p = 9e-86)<br><br>Cell junction assembly (p = 9e-74)<br><br>Somatic stem cell maintenance (p = 6e-65)	Somatic stem cell maintenance (p = 5e-100)<br><br>Mesoderm morphogenesis (p = 2e-63)<br><br>Cell junction assembly (p = 6e-61)
MSigDB Perturbation (related to ES cells only)	Genes with DNA sequences bound by RARA and RARG in ES cells (p = 0)<br><br>Genes down-regulated in ES cells (embryonic stem) heterozygotic for KDM1A loss of function mutant compared to the homozygotic loss of the gene (p=2e-186)<br><br>Genes with low-CpG-density promoters (LCP) bearing histone H3 trimethylation mark at K4 (H3K4me3) in embryonic stem cells (ES) (p=1e-120)	Genes with DNA sequences bound by RARA and RARG in ES cells (p = 1e-298)<br><br>Genes down-regulated in ES cells (embryonic stem) heterozygotic for KDM1A loss of function mutant compared to the homozygotic loss of the gene (p=4e-128)<br><br>Genes with low-CpG-density promoters (LCP) bearing histone H3 trimethylation mark at K4 (H3K4me3) in embryonic stem cells (ES) (p=1e-81)

Additional file 2: SCL-exo identification of 5hmCpGs in mouse ESCs. (**a**) Sequencing statistics for the three technical replicates of SCL-exo in E14 mESCs. SCL-exo id CpGs indicate reads with a single CpG within 10 bases from their start. Probable CpGs indicate reads with at least two CpGs within 10 bases from their start. Possible CpHs indicate reads with at least one C not followed by a G within 10 bases from their start. (**b**) IGB visualization of hMeDIP and SCL-exo signals at id CpGs from technical replicates 1, 2 and 3, and from the consensus .wig file in a region of chr11. (**c**) Graphical representation of the number of CpGs identified by running the SCL-exo CpG identification algorithm either on the Input-seq or on the SCL-exo reads, as a function of coverage. (**d**) IGB visualization of hMeDIP signal and 5hmCpGs identified either by TAB-seq (.bed file of overlapping CpGs from two technical replicates with at least 20% hydroxymethylation), or by SCL-exo (.bed file of CpGs identified in at least 2 out of 3 technical replicates and with a coverage ≥ 40x), within *Ranbp17* on chr11. (**e**) Functional annotation of the SCL-exo and TAB-seq identified 5hmCpGs included in hMeDIP peaks. Annotation was done with GREAT and binomial raw *p* values are given in brackets for each item.
