# Supplementary material for: Single-CpG resolution mapping of 5-hydroxymethylcytosine by chemical labeling and exonuclease digestion identifies evolutionarily unconserved CpGs as TET targets
Source: Genome Biol. 2016 Mar 29;17:56. doi: 10.1186/s13059-016-0919-y (PMC4810514; doi:10.1186/s13059-016-0919-y)
Supplement: Additional file 3: — Is a figure including additional information on the conservation status of hydroxymethylated CpG and their genome wide distribution and inclusion in H3K4me1-marked regions. Additional file 3 complements Fig. 6. (PDF 500 kb) [file 13059_2016_919_MOESM3_ESM.pdf]

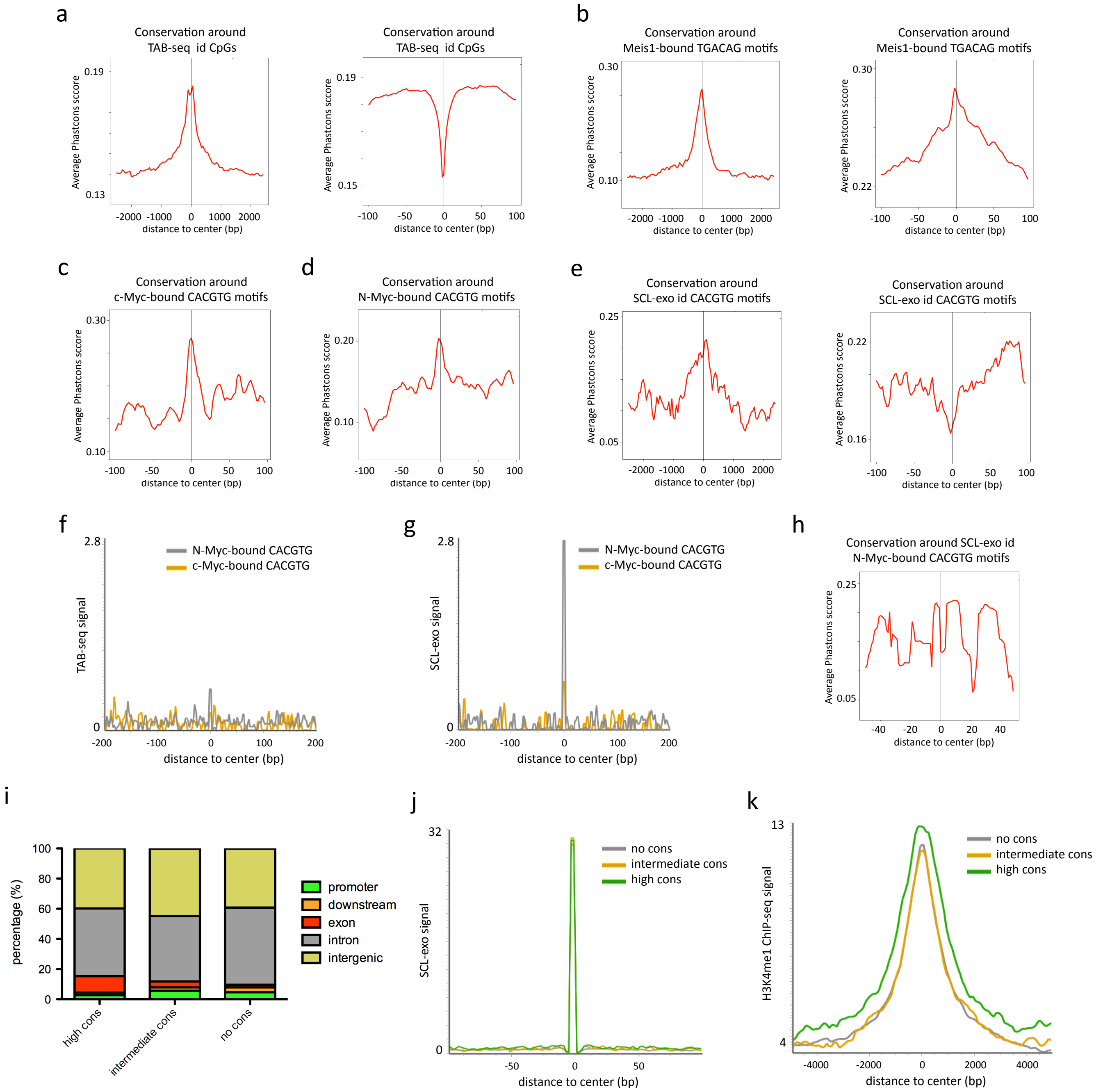

Additional file 3: Lack of conservation of SCL-exo id CpGs among vertebrates. (**a**) Average PhastCons score around TAB-seq id CpGs with at least 20% of hydroxymethylation in mouse ES cells, either in a 5,000 bp window (left panel) or in a 200 bp window (right panel). (**b**) Average PhastCons score around Meis1 bound TGACAG motifs either in a 5,000 bp window (left panel) or in a 200 bp window (right panel). (**c**) Average PhastCons score around c-Myc bound CACGTG sites in mouse ES cells. (**d**) Average PhastCons score around N-Myc bound CACGTG sites (n = 323) in mouse ES cells. (**e**) Average PhastCons score around CACGTG sites overlapping with SCL-exo id CpGs either in a 5,000 bp window (left panel) or in a 200 bp window (right panel). (**f**) TAB-seq signal at N-Myc- and c-Myc-bound CACGTG sites in ES cells. (**g**) SCL-exo signal at N-Myc- and c-Myc-bound CACGTG sites in ES cells. (**i**) Average PhastCons score around N-Myc bound CACGTG sites with SCL-exo signal (n = 14) in mouse ES cells(**i**) Gene-centered annotation of SCL-exo id CpGs (NPLCs) sorted according to their conservation (cons). (**j**) Average profile of SCL-exo signal at SCL-exo id CpGs (NPLcs) sorted according to their conservation. (**k**) Average profile of H3K4me1 ChIP-seq signal at SCL-exo id CpGs (NPLCs) sorted according to their conservation.
